# Pyridoxal phosphate synthases PdxS/PdxT are required for *Actinobacillus pleuropneumoniae* viability, stress tolerance and virulence

**DOI:** 10.1371/journal.pone.0176374

**Published:** 2017-04-27

**Authors:** Fang Xie, Gang Li, Yalei Wang, Yanhe Zhang, Long Zhou, Chengcheng Wang, Shuanghong Liu, Siguo Liu, Chunlai Wang

**Affiliations:** 1 State Key Laboratory of Veterinary Biotechnology, Division of Bacterial Diseases, Harbin Veterinary Research Institute, Chinese Academy of Agricultural Sciences, Harbin, People’s Republic of China; 2 Shanghai Veterinary Research Institute, Chinese Academy of Agricultural Sciences, Shanghai, People’s Republic of China; University of Cape Town, SOUTH AFRICA

## Abstract

Pyridoxal 5’-phosphate (PLP) is an essential cofactor for numerous enzymes involved in a diversity of cellular processes in living organisms. Previous analysis of the *Actinobacillus pleuropneumoniae* S-8 genome sequence revealed the presence of *pdxS* and *pdxT* genes, which are implicated in deoxyxylulose 5-phosphate (DXP)-independent pathway of PLP biosynthesis; however, little is known about their roles in *A*. *pleuropneumoniae* pathogenicity. Our data demonstrated that *A*. *pleuropneumoniae* could synthesize PLP by PdxS and PdxT enzymes. Disruption of the *pdxS* and *pdxT* genes rendered the pathogen auxotrophic for PLP, and the defective growth as a result of these mutants was chemically compensated by the addition of PLP, suggesting the importance of PLP production for *A*. *pleuropneumoniae* growth and viability. Additionally, the *pdxS* and *pdxT* deletion mutants displayed morphological defects as indicated by irregular and aberrant shapes in the absence of PLP. The reduced growth of the *pdxS* and *pdxT* deletion mutants under osmotic and oxidative stress conditions suggests that the PLP synthases PdxS/PdxT are associated with the stress tolerance of *A*. *pleuropneumoniae*. Furthermore, disruption of the PLP biosynthesis pathway led to reduced colonization and attenuated virulence of *A*. *pleuropneumoniae* in the BALB/c mouse model. The data presented in this study reveal the critical role of PLP synthases PdxS/PdxT in viability, stress tolerance, and virulence of *A*. *pleuropneumoniae*.

## Introduction

Vitamin B6 is an essential cofactor in a multitude of cellular enzymatic reactions and is required in the metabolism of carbohydrates, amino acids, and fatty acids [1]. Pyridoxal 5’-phosphate (PLP) is the biochemically active form of vitamin B6 [2]. To date, studies have identified more than 160 enzymes that are functionally dependent on PLP [3, 4]. Vitamin B6 plays an important role in living organisms, and humans and animals must obtain it from food. By contrast, bacteria, fungi, and plants are able to synthesize PLP via *de novo* pathways.

**Fig 1:**
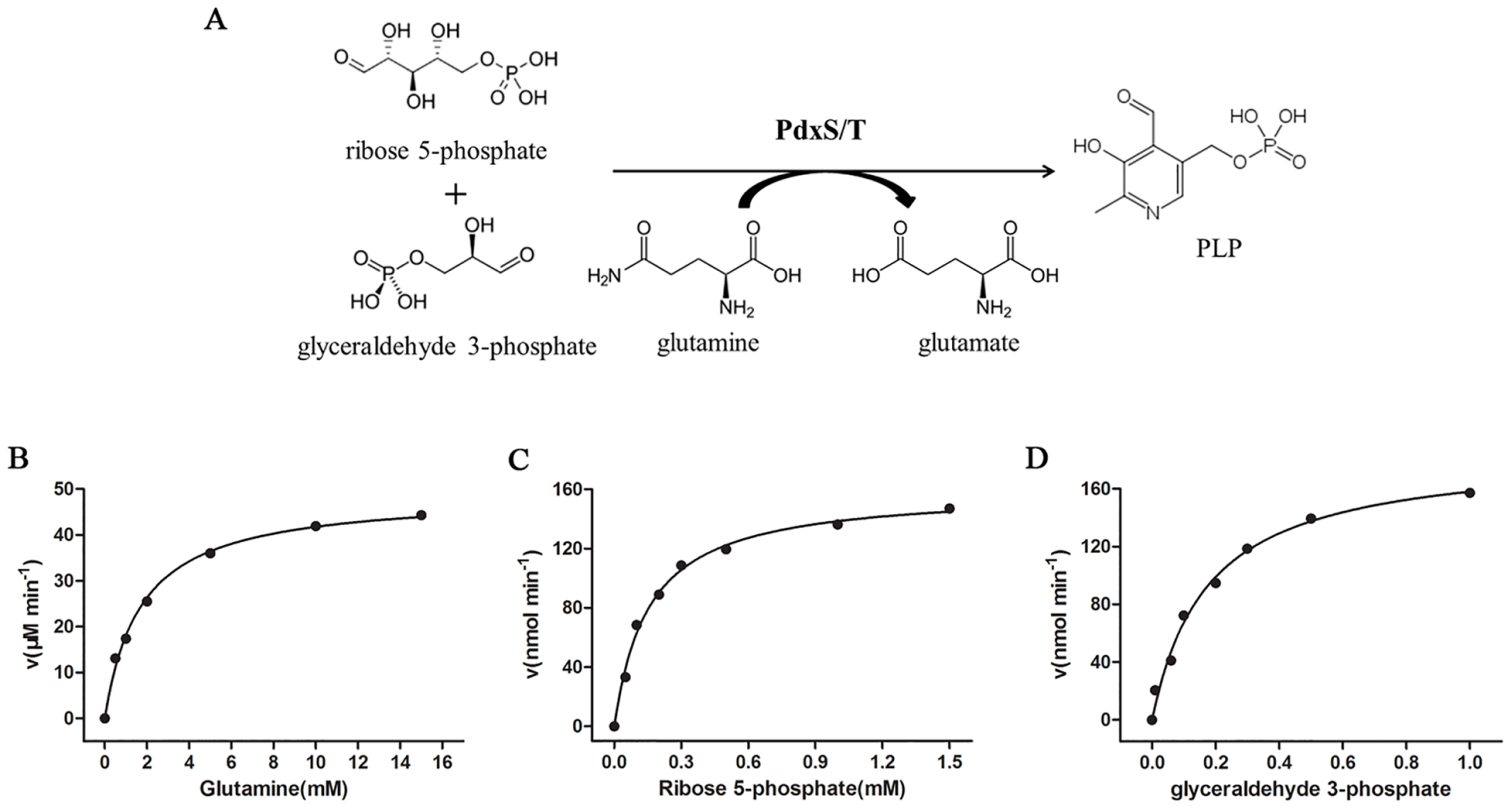
Enzymatic activity of PdxS and PdxT. (A) Substrates and product of a typical PLP synthase. (B) Effect of glutamine (0–15 mM) on glutaminase activity of PdxS and PdxT. (C) Effect of ribose 5-phosphate (0–1.5 mM) on PLP synthesis of PdxS and PdxT. (D) Effect of glyceraldehyde 3-phosphate (0–1.0 mM) on PLP synthesis of PdxS and PdxT. The kinetic constants were estimated by fitting to the Michaelis-Menten equation.

Bacteria possess two distinct *de novo* PLP biosynthesis pathways, referred to as deoxyxylulose 5-phosphate (DXP)-dependent pathway and DXP-independent pathway [5, 6]. *Escherichia coli* and other members of the γ-subdivision of proteobacteria adopt the DXP-dependent pathway, which involves two enzymes PdxA and PdxJ. In contrast, the DXP-independent pathway is found in most eubacteria, fungi, protozoa, archaea, plants and metazoan, and requires PdxS and PdxT enzymes [5, 7]. As shown in Fig 1A, by converting glutamine into glutamate, PdxT generates ammonia, which is used by PdxS to synthesize PLP from a 5 and a 3 carbon sugar, such as ribose 5-phosphate and glyceraldehyde 3-phosphate [8, 9]. Although the *de novo* PLP biosynthesis pathways have been found in non-pathogenic bacteria, such as *Escherichia coli* and *Bacillus subtilis* [5, 10], the relation between PLP biosynthesis pathways and bacterial pathogenicity remains poorly understood. However, a previous study on *Mycobacterium tuberculosis* suggested that PLP biosynthesis was also essential for bacterial survival and virulence [8].

*Actinobacillus pleuropneumoniae* is a Gram-negative bacterial pathogen responsible for porcine pleuropneumonia, which is a severely contagious respiratory disease that causes major economic losses for the swine industry worldwide [11]. Effective survival and persistence in pigs is a critical hindrance for *A*. *pleuropneumoniae* eradication [11, 12]. Recent analysis of the *A*. *pleuropneumoniae* S-8 genome sequence revealed the presence of the *pdxS* and *pdxT* genes [13]. Additionally, a previous study revealed that both *pdxS* and *pdxT* genes were downregulated after inactivation of the *clpP* gene which is required for stress tolerance in *A*. *pleuropneumoniae* [14].

To date, the vitamin B6 biosynthesis pathway has not been characterized in *A*. *pleuropneumoniae*. Furthermore, whether *A*. *pleuropneumoniae* adopts the DXP-independent pathway or whether the PLP synthases PdxS/PdxT are required for viability, stress tolerance, and virulence of *A*. *pleuropneumoniae* remain unclear. In the present study, we identified and characterized the function of enzymes PdxS and PdxT in the vitamin B6 biosynthesis pathway in *A*. *pleuropneumoniae*. We constructed knockouts in the *pdxS* and *pdxT* genes, respectively and investigated the role of PdxS and PdxT in viability, stress tolerance, and virulence of *A*. *pleuropneumoniae*.

## Materials and methods

### Ethics statement

Animal experiments were approved by Animal Ethics Committee of Harbin Veterinary Research Institute of the Chinese Academy of Agricultural Sciences (CAAS) and carried out in strict accordance with animal ethics guidelines and approved protocols (Heilongjiang-SYXK-2011–022). All efforts were made to minimize animal suffering.

### Bacterial strains and growth conditions

The bacterial strains, plasmids, and primers used in this study are described in S1 and S2 Tables. The *A*. *pleuropneumoniae* strains were cultured in a brain heart infusion (BHI) medium supplemented with 10 μg/mL nicotinamide dinucleotide (NAD) (Sigma-Aldrich, USA). For the culture of *A*. *pleuropneumoniae* transconjugants (single crossovers), BHI medium was supplemented with 10 μg/mL of NAD and 7 μg/mL of chloramphenicol. *E*. *coli* β2155 was grown in Luria-Bertani (LB) medium supplemented with 1 mM diaminopimelic acid (DAP) (Sigma-Aldrich, USA). The chemically defined medium (CDM) was prepared as previously described [15], without the addition of NH_4_Cl. All strains were routinely grown at 37°C.

### Protein expression and purification

The coding sequences of *A*. *pleuropneumoniae pdxS* and *pdxT* genes were PCR-amplified from S-8 genomic DNA using specific primers SF/SR and TF/TR (S1 Table). The digested PCR products were ligated with NdeI/XhoI-digested pET22b(+) (Novagen). The recombinant plasmids were confirmed by sequencing and used to transform into *E*. *coli* BL21 (DE3). The expression of the each target protein was induced for 18 h at 16°C with 0.5 mM isopropyl 1-thio-β-D-galactopyranoside (IPTG) in LB broth containing 50 μg/ml ampicillin. The His6-tag fusion proteins were loaded onto a Ni Sepharose 6 Fast Flow column (GE Healthcare, United States) and purified as previously described [16]. The recombinant protein concentrations were determined using a bicinchoninic acid (BCA) protein assay kit (Beyotime, China).

### Glutaminase activity assay

Glutaminase activity was measured as described previously [17]. Samples of 8 μM PdxS, 8 μM PdxT, or 8 μM mixture of both proteins was incubated with 10 mM glutamine, 6 units of bovine glutamate dehydrogenase, and 0.5 mM 3-acetylpyridine adenine dinucleotide (APAD; Sigma) in a total reaction volume of 300 μl of 50 mM Tris-Cl (pH 8.0). The reaction mixture was incubated at 37°C. The absorbance of each sample was read at a wavelength of 363 nm (OD_363_).

### PLP formation assay

PLP formation assay was performed using a procedure as previously described [17]. Samples of 8 μM PdxS, 8 μM PdxT, or 8 μM mixture of both proteins was incubated with 0.5 mM ribose 5-phosphate, 1mM DL-glyceraldehyde 3-phosphate, 10 mM glutamine or 10 mM ammonium sulfate was added to a final volume of 300μl of 50 mM Tris-HCl (pH 8.0). The reaction mixture was incubated at 37°C. The absorbance of each sample was read at a wavelength of 414 nm (OD_414_).

### Construction of gene deletion mutants and complemented strains

**Fig 2:**
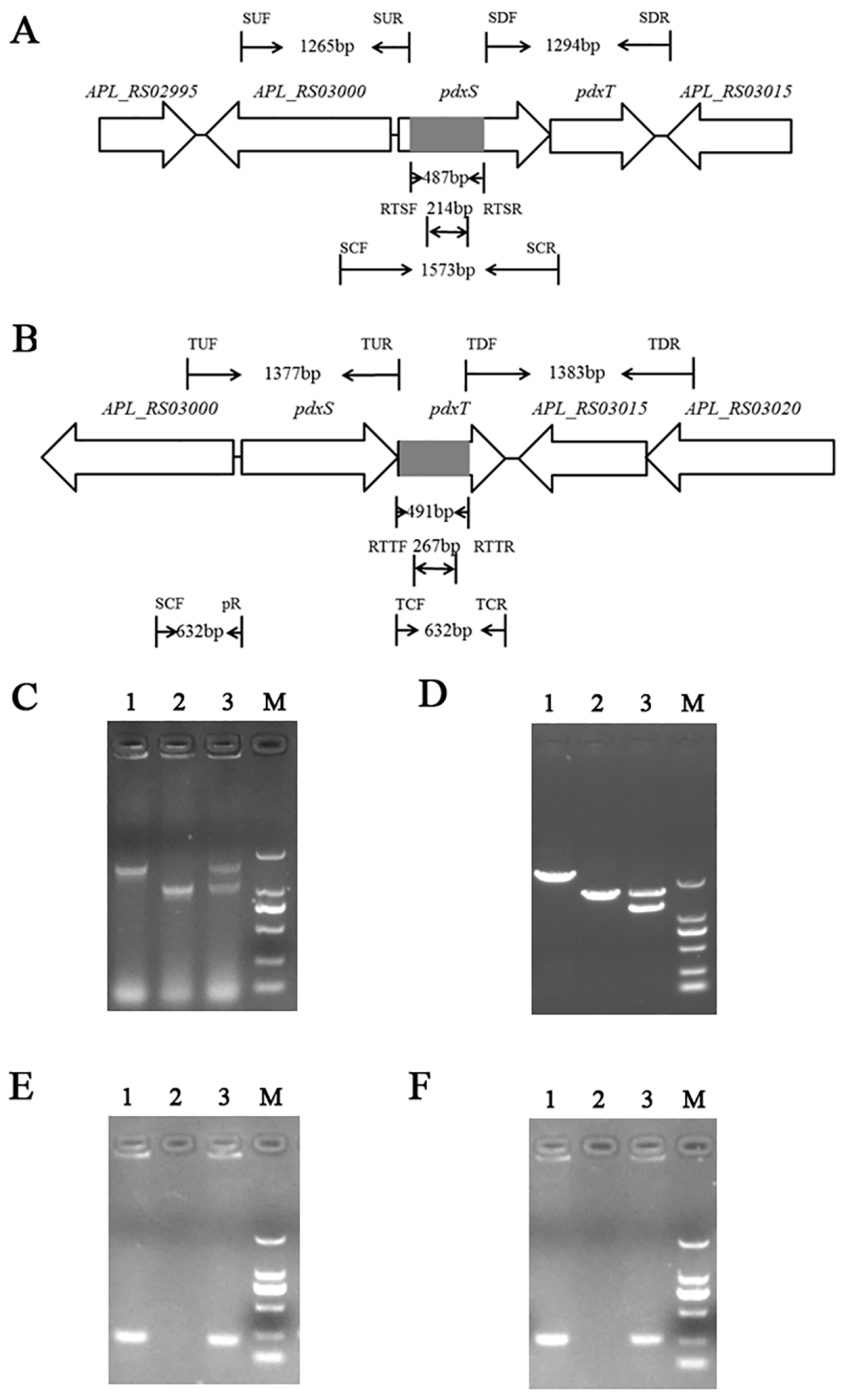
Chromosomal inactivation of *the pdxS* and *pdxT* genes. (A) Schematic representation of the *A*. *pleuropneumoniae pdxS* locus. The figure shows the binding locations for the oligonucleotide primers used to amplify the two flanking regions (1265 bp and 1294 bp, respectively) used in the construction of the pEM△*pdxS* plasmid and the diagnostic PCR analysis of the S-8Δ*pdxS* mutant (1106 bp) and WT S-8 strains (1573 bp). The S-8Δ*pdxS* mutant contains a 487 bp deletion (shadowed domain) in the *pdxS* gene. (B) (A) Schematic representation of the *A*. *pleuropneumoniae pdxT* locus. The figure shows the binding locations for the oligonucleotide primers used to amplify the two flanking regions (1377 bp and 1387 bp, respectively) used in the construction of the pEM△*pdxT* plasmid and the diagnostic PCR analysis of the S-8Δ*pdxT* mutant (660 bp) and WT S-8 strains (1151 bp). The S-8Δ*pdxT* mutant contains a 491 bp deletion (shadowed domain) in the *pdxT* gene. (C) PCR identification of the S-8Δ*pdxS* mutant using the primers SCF/SCR. For lane 1, the WT S-8 strain (1573 bp); for lane 2, the S-8Δ*pdxS* mutant (1106 bp); for lane 3, the complemented S-8Δ*pdxS*comp strain (1573 and 1106 bp). (D) PCR identification of the S-8Δ*pdxT* mutant using the primers SCF/TCR. For lane 1, the WT S-8 strain (2151bp); for lane 2, the S-8Δ*pdxT* mutant (1660 bp); for lane 3, the complemented S-8Δ*pdxT*comp strain (1660 and 1264 bp). (E) PCR identification of the S-8Δ*pdxS* mutant using the primers RTSF/RTSR. For lane 1, the WT S-8 strain (214 bp); for lane 2, the S-8Δ*pdxS* mutant; for lane 3, the complemented S-8Δ*pdxS*comp strain (214 bp). (F) PCR identification of the S-8Δ*pdxT* mutant using the primers RTTF/RTTR. For lane 1, the WT S-8 strain (267 bp); for lane 2, the S-8Δ*pdxT* mutant; for lane 3, the complemented S-8Δ*pdxT*comp strain (267 bp). For lane M, DL2000 DNA marker (from top to bottom: 2000, 1000, 750, 500, 250, and 100 bp).

The primers used for the construction of the deletion mutants S-8*ΔpdxS* and S-8*ΔpdxT* are listed in S1 Table. Primers SUF/SUR and SDF/SDR were used to amplify the two segments flanking with the *pdxS* gene. Using single-overlap extension PCR (SOE PCR), the fragment with a 487 bp internal deletion in the *pdxS* gene (from nt 54 to 539) was generated, and cloned into the conjugative vector pEMOC2 [18] to construct plasmid pEM△*pdxS*. Using *E*. *coli* β2155 and a single-step transconjugation system [19, 20], plasmid pEM△*pdxS* was applied to introduce the *pdxS* mutation into the S-8 strain. After two homologous recombination steps, the *A*. *pleuropneumoniae* S-8*ΔpdxS* mutant was verified by sequencing and PCR analyses using primers SCF/SCR and RTSF/RTSR (Fig 2A).

The deletion of *pdxT* gene in *A*. *pleuropneumoniae* S-8 was performed using the same procedure as described above. The primers TUF/TUR, and TDF/TDR were used to generate a 491 bp internal deletion in the *pdxT* gene (from nt 7 to 497), and the S-8*ΔpdxT* mutant was confirmed by PCR analyses with the primers SCF/TCR and RTTF/RTTR (Fig 2B).

The promoter of PdxS and PdxT was predicted using the BPROM program (http://www.softberry.com/). The pLpdxS plasmid was constructed by cloning the 1573-bp PCR product amplified with the primers SCF/SCR (S1 Table), which contained the entire *pdxS* open reading frame (ORF) and the upstream region to include the native promoter, into plasmid pLS88 [21]. Using SOE PCR, the 1264-bp fragment containing the upstream promoter and entire *pdxT* ORF was generated, and ligated into the plasmid pLS88 to yield plasmid pLpdxT plasmid (Fig 2A and 2B). The plasmids pLpdxS and pLpdxT were electroporated into S-8*ΔpdxS* and S-8*Δpdx*T, respectively for *trans* complementation. The complemented strains were selected on BHI agar containing 20 μg/mL of kanamycin, and were verified by PCR using the primers SCF/SCR or TCF/TCR.

### RNA isolation and qRT-PCR

For RNA isolation, *A*. *pleuropneumoniae* strains were grown to mid-logarithmic phase in 3 ml of BHI medium. The cultures were harvested by centrifugation at 10,000 *g* at 4°C. Total RNA was extracted using RNeasy kit (Qiagen) and complementary DNA (cDNA) was synthesized using the PrimeScript RT reagent kit (TaKaRa, Japan) according to the manufacturer's instructions. The primers used for analysis of gene expression are listed in S2 Table. The cDNA samples were amplified using SYBR Green I (TakaRa). Real-time polymerase chain reactions were performed in a MicroAmp Optical 96-well reaction plate using qTOWER 2.2 system (Analytikjena, Germany). The quantitative qRT-PCR experiments were performed in triplicate, with *recF* as an internal control [22].

### *In vitro* growth assays

*In vitro* growth assays were conducted as previously described [23]. The *A*. *pleuropneumoniae* wild-type (WT) S-8, S-8Δ*pdxS* mutant, S-8Δ*pdxT* mutant and complemented strains S-8Δ*pdxS*comp and S-8Δ*pdxT*comp were grown in 3 ml of BHI medium supplemented with 10 μM PLP for 18 h, then washed three times with CDM medium to remove residual PLP and diluted to optical densities at 600 nm (OD_600_) of 0.1. The fresh cultures in 10 ml of CDM medium were respectively supplemented with 10 μM PLP (Sigma), 1 mM ammonium sulfate or not supplemented any chemicals. The cultures were then incubated at 37°C. The OD_600_ values were recorded at an interval of 2 h using the Eppendorf BioPhotometer (Eppendorf, Germany).

### *In vitro* stress assays

*In vitro* stress assays were performed as described previously [14]. The *A*. *pleuropneumoniae* WT S-8, S-8Δ*pdxS* mutant, S-8Δ*pdxT* mutant and complemented strains S-8Δ*pdxS*comp and S-8Δ*pdxT*comp were grown to OD_600_ 0.8 in BHI medium supplemented with 10 μM PLP. We inoculated bacteria at different time points according to the growth curves to try to get all strains to achieve the OD600 of 0.8 at the same time. In addition, we inoculated bacteria at an interval of 0.5 h to make sure that. Cells (10^8^ CFU/mL) of each strain from the broth cultures were washed three times with un-supplemented BHI medium, and harvested by centrifugation at 4000 g for 10 min. To test the tolerance of the cells to oxidative stress, cells of each strain were resuspended in 1 ml of BHI medium containing 5 mM H_2_O_2_ and incubated in the presence or absence of PLP for 45 min at 37°C. To test the tolerance of the cells to osmotic stress, cells of each strain were resuspended in 1 ml of BHI medium containing 0.4 M NaCl and incubated in the presence or absence of PLP for 45 min at 37°C. Cells of each strain were resuspended in BHI medium in the presence or absence of PLP as controls and incubated for 45 min at 37°C. All of the cultures of each stress assay were washed three times with BHI medium to remove residual H_2_O_2_ or NaCl, and serially diluted and cultured on BHI agar plates supplemented with 10 μM PLP. The cell count was determined after 24 h of incubation. Values of stress resistance were calculated as [(stressed sample CFU/ ml)/ (control sample CFU/ ml)]×100.

### Transmission electron microscopy

The *A*. *pleuropneumoniae* WT S-8, S-8Δ*pdxS* mutant, S-8Δ*pdxT* mutant and complemented strains S-8Δ*pdxS*comp and S-8Δ*pdxT*comp were cultivated in BHI medium supplemented with 10 μM PLP at 37°C for 16 h, then washed three times with un-supplemented BHI medium to remove residual PLP and diluted to optical densities at 600 nm (OD_600_) of 0.05. The fresh cultures were grown to mid-exponential growth phase in 10 ml of BHI medium with or without 10 μM PLP. The cells were washed three times with PBS and fixed for 2 h in 2% osmic acid. Dehydration was performed in upgraded ethanol, and then the samples were embedded in SPI-Pon 812 resin (emicron) for two days. Resin-soaked sample blocks were polymerized at 70°C, and samples were counterstained with uranyl acetate and lead citrate. Electron micrographs were recorded with a transmission electron microscope (JEM-1200EX, JEOL, Japan).

### Mouse *in vivo* experiments

BALB/c mouse has been acknowledged as an appropriate model for *A*. *pleuropneumoniae* infection [24, 25]. Specific-pathogen-free, six-week-old female BALB/c mice (Beijing Vital River Laboratory Animal Co., Ltd.) were used for virulence evaluation of *A*. *pleuropneumoniae* WT S-8, S-8Δ*pdxS* mutant, and S-8Δ*pdxT* mutant strains. Briefly, all *A*. *pleuropneumoniae* strains were cultured in BHI medium supplemented with 10 μM PLP at 37°C, and harvested during the mid-exponential phase and washed three times with sterile PBS. A total of 160 mice were randomly divided into 16 groups (n = 10/group). For each strain, five experimental groups were inoculated intraperitoneally with 100 μL of PBS containing varying concentrations of bacterial suspension (10^5^–10^9^ CFU). Non-infected mice in the control group were inoculated with 100 μL of sterile PBS (pH 7.4). Animal suffering was reduced with buprenorphine (0.05 mg/kg), given subcutaneously for analgesia every 12 hours during the first 72 hours after infection. The health status and the weight of the mice were monitored twice daily for a 14-day period and humane endpoints used to determine if the mice met criteria to be euthanized [26]. These criteria included weight loss >10–15%, lethargy, inability to stand, anorexia or flocked together for more than 6 hours. Mice meeting criteria were euthanized by cervical dislocation under isoflurane anesthesia. The 50% lethal dose (LD_50_) of *A*. *pleuropneumoniae* WT strain S-8, S-8Δ*pdxS* mutant, and S-8Δ*pdxT* mutant strains were calculated as previously described by Reed-Muench [27].

### Enumeration of bacterial load in organs

A total of 15 specific-pathogen-free, six week-old female BALB/c mice were randomly divided into 3 groups (n = 5), and each group was intraperitoneally administered with 1.0×10^7^ CFU of WT S-8 strain, S-8Δ*pdxS* mutant, and S-8Δ*pdxT* mutant strains respectively. Three (3) days post-infection, mice from each group were humanely euthanized and the organs of lung, liver, and kidney were removed aseptically. Samples were weighed, and homogenized using a tissue homogenizer (100 mg weight/ml of PBS). Viable counts in serial dilutions of homogenates were determined following culture on BHI agar plates for 24 h at 37°C.

### Statistical analysis

All statistical analyses were performed using GraphPad Prism version 5.01 (GraphPad Software Inc., USA). The data are expressed as the means +/- standard deviation. *P*-values less than 0.05 were considered statistically significant.

## Results

### PdxS and PdxT are involved in PLP synthesis in *A*. *pleuropneumoniae*

The ORFs of *pdxS* and *pdxT* genes are 888-bp and 576-bp in length, respectively, and encode the putative pyridoxal 5'-phosphate synthase subunits PdxS and PdxT. Searching the respective conserved domains in NCBI database revealed that PdxS has SOR_SNZ domain which represents a family of pyridoxal 5'-phosphate synthase and PdxT has SNO domain which represents glutamine amidotransferase family. Both PdxS and PdxT shared a high identity in the amino acid sequences with their orthologous sequences in *Bacillus subtilis* (84 and 55%, respectively) and *Streptococcus pneumoniae* (64 and 41%, respectively). In this study, to determine the activities of PdxS and PdxT in vitamin B6 formation *in vitro*, *A*. *pleuropneumoniae pdxS* and *pdxT* genes were overexpressed in *E*. *coli* BL21 (DE3) and the recombinant His6-tag fusion proteins were purified using Ni^2+^-affinity chromatography. SDS—PAGE analysis showed that the recombinant proteins, with the molecular weight of approximately 35.3 kD and 23.4 kD (S1 Fig), respectively, were obtained when the cells were induced with 0.5 mM IPTG at 16°C for 18 h.

Glutaminase activity of PdxT was only detected in the presence of its partner protein PdxS. As shown in Fig 1B, varying the concentration of glutamine in the assay produced typical Michaelis-Menten kinetics, the catalytic constants *K*_M_ and *k*_cat_ were 1.72±0.14 mM and 6.12±0.14 min^-1^ respectively. Furthermore, the ribose 5-phosphate and glyceraldehyde 3-phosphate were served as substrates of PLP synthase PdxS and PdxT, and tested for their ability to form PLP. The kinetic constants were determined as *K*_M_ = 159.8±13.5 μM, *k*_cat_ = 0.020±0.0005 min^-1^ for ribose 5-phosphate (Fig 1C), and *K*_M_ = 175.7±23.0 μM, *k*_cat_ = 0.023±0.0011 min^-1^ for glyceraldehyde 3-phosphate, respectively (Fig 1D).

### *A*. *pleuropneumoniae* S-8Δ*pdxS* and S-8Δ*pdxT* mutants are auxotrophic for PLP

To demonstrate the function of PLP biosynthesis in *A*. *pleuropneumoniae*, *pdxS* and *pdxT*-knockout mutants, S-8Δ*pdxS* and S-8Δ*pdxT*, respectively, were generated using the double-crossover homologous recombination approach and confirmed by PCR and sequencing (Fig 2). The results of qRT-PCR showed that the transcription levels of the downstream genes were unaffected, confirming that the mutations in S-8Δ*pdxS* and S-8Δ*pdxT* strains were nonpolar (S2 Fig). Complementation of the S-8Δ*pdxS* and S-8Δ*pdxT* were achieved using the plasmid pLpdxS and pLpdxT, respectively (Fig 2). The complemented mutants were designated S-8Δ*pdxS*comp and S-8Δ*pdxT*comp. The results of qRT-PCR showed that the transcriptions of *pdxS* in S-8Δ*pdxS* and *pdxT* in S-8Δ*pdxT* were virtually undetectable and partially restored in the complemented strains.

**Fig 3:**
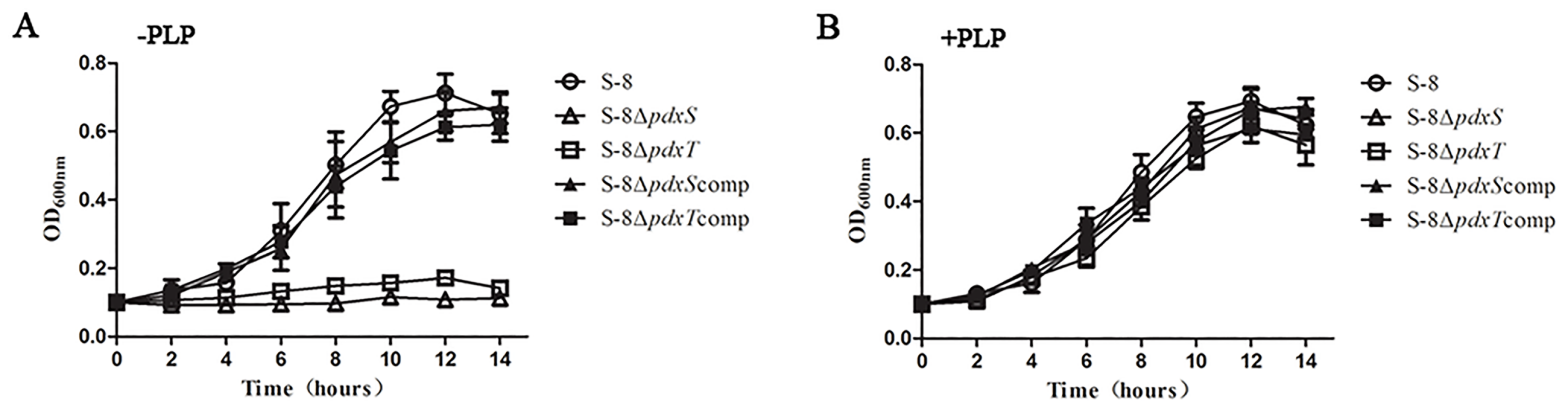
Growth characteristics of the *A*. *pleuropneumoniae* strains. The growth curves of the WT S-8, S-8Δ*pdxS*, S-8Δ*pdxT*, S-8Δ*pdxS*comp strain and S-8Δ*pdxT*comp strains in the absence (A) or presence (B) of PLP. Overnight culture of each strain was inoculated into fresh CDM medium with or without 10μM PLP and grown at 37°C for 10 h. Growth was monitored by OD_600_ at an interval of 2 h.

The growth properties of the *A*. *pleuropneumoniae* WT S-8, S-8Δ*pdxS*, S-8Δ*pdxT*, S-8Δ*pdxS*comp, and S-8Δ*pdxT*comp strains were investigated *in vitro*. Upon cultivation in the absence of PLP, both S-8Δ*pdxS* and S-8Δ*pdxT* mutants exhibited severe growth defects (Fig 3A). It is suggested that PLP is essential for *A*. *pleuropneumoniae* growth *in vitro*. We therefore analyzed the growth of S-8Δ*pdxS* and S-8Δ*pdxT* mutants in the presence PLP supplementation, the defective growth of these mutants could be recovered, fundamentally similar to growth curves of the WT strain S-8 (Fig 3B). The growth curves of the S-8, S-8Δ*pdxS*comp, and S-8Δ*pdxT*comp strains were similar in the absence and presence of PLP. These data suggest critical roles for PdxS and PdxT proteins in *A*. *pleuropneumoniae* PLP biosynthesis.

### The PLP synthesis activity of PdxS is not dependent on PdxT in the presence of ammonium

**Fig 4:**
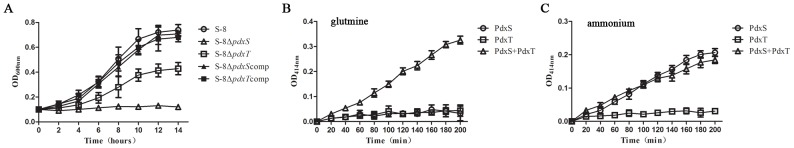
PLP synthase activity of PdxS in the presence of ammonium. (A) The growth curves of the WT S-8, S-8Δ*pdxS*, S-8Δ*pdxT* mutant, S-8Δ*pdxS*comp strain and S-8Δ*pdxT*comp strains in the presence of ammonium supplementation. Overnight culture of each strain was diluted into fresh CDM medium supplemented with 1mM ammonium sulfate. Bacteria were grown at 37°C for 10 h and growth was monitored by OD_600_ at an interval of 2h. (B) PLP synthase activity of PdxS, PdxT, and the mixture of the two proteins using glutamine as substrate in the reaction. (C) PLP synthase activity of PdxS, PdxT, and the mixture of the two proteins using ammonium substitute for glutamine in the reaction. Points indicate the mean values of three independent assays, and error bars indicate standard deviations.

The growth properties of the *A*. *pleuropneumoniae* WT S-8, S-8Δ*pdxS* mutant, S-8Δ*pdxT* mutant and complemented strains S-8Δ*pdxS*comp and S-8Δ*pdxT*comp were also analyzed in the presence of ammonium supplementation *in vitro*. With ammonium sulfate supplementation, the defective growth rate of S-8Δ*pdxT* mutant was partially restored, but the defective growth of the S-8Δ*pdxS* mutant remained unchanged (Fig 4A). We then examined PLP synthesis activity of PdxS using ammonium substitute for glutamine in the reaction. Unlike using glutamine as substrate, the individual PdxS could produce PLP in the addition of ammonium (Fig 4B and 4C). The PLP synthase had a specific activity of 996 ± 54 pmol min^-1^ mg^-1^ using glutamine as substrate, and of 508 ± 38 pmol min^-1^ mg^-1^ using ammonium sulphate as substrate.

### *A*. *pleuropneumoniae* S-8Δ*pdxS* and S-8Δ*pdxT* mutants are sensitive to NaCl and H_2_O_2_

**Fig 5:**
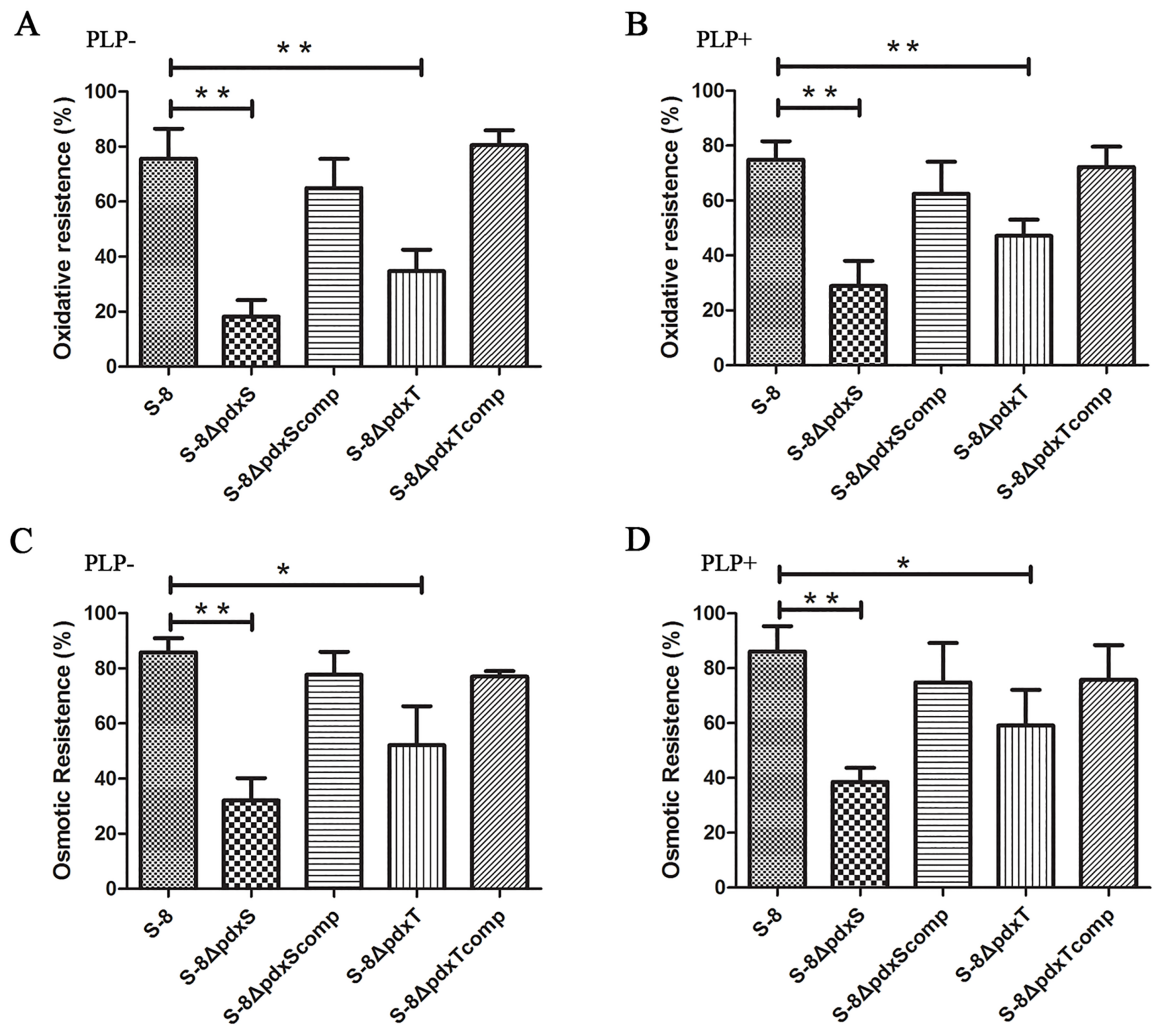
Response of *A*. *pleuropneumoniae* mutants to oxidative and osmotic stresses. Overnight culture of WT S-8, S-8*ΔpdxS*, S-8*ΔpdxT*, S-8Δ*pdxS*comp strain and S-8Δ*pdxT*comp strains were diluted into fresh BHI broth and grown to OD_600_ 0.8. Bacteria were then treated with 5 mM H_2_O_2_ in the absence (A) and presence (B) of PLP for 45 min, 0.4 M NaCl in the absence (C) and presence (D) of PLP for 45 min. Values of stress resistance were calculated as [(stressed sample CFU/ ml)/ (control sample CFU/ ml)]×100. The data shown are the means of three independent assays, and error bars indicate standard deviations. **, *p* < 0.01; *, *p* < 0.05.

The viability of the WT S-8, S-8Δ*pdxS* mutant, S-8Δ*pdxT* mutant and complemented strains S-8Δ*pdxS*comp and S-8Δ*pdxT*comp were investigated when exposed to oxidative stress and osmotic stress conditions. As shown in S3A Fig, in the absence of PLP and when the cells were exposed to H_2_O_2_-induced oxidative stress, the viable counts of S-8Δ*pdxS* and S-8Δ*pdxT* were much lower than that of the S8 cells. The survival rates of the S-8Δ*pdxS* mutant and S-8Δ*pdxT* mutant were 18.19% and 34.73%, respectively, which were significantly lower (*P* < 0.01) than that of WT S-8 (75.68%) (Fig 5A). To exclude the influence of growth defects of S-8Δ*pdxS* and S-8Δ*pdxT* in the absence of PLP, the cells of each strain were also exposed to oxidative stress in the presence of PLP supplementation. The viable counts of S-8Δ*pdxS* and S-8Δ*pdxT* were still lower than that of the S8 cells, although increased compared to the viable counts of S-8Δ*pdxS* and S-8Δ*pdxT* exposed to oxidative stress in the absence of PLP (S3B Fig). As shown in Fig 5B, in the presence of PLP supplementation, 74.86% of WT S-8 survived. However, S-8Δ*pdxS* and S-8Δ*pdxT* exhibited 28.90% (*P* < 0.01) and 47.16% (*P* < 0.01) survival rates, respectively. Similar results were obtained when the cells were exposed to osmotic stress. The viable counts of S-8Δ*pdxS* and S-8Δ*pdxT* were lower than that of the S8 cells (S3C and S3D Fig). In the absence of PLP, WT S-8 cells under osmotic stress exhibited the 85.89% survival rate, whereas the S-8Δ*pdxS* mutant and the S-8Δ*pdxT* mutant respectively exhibited 32.08% (*P* < 0.01) and 52.13% (*P* < 0.05) survival rate (Fig 5C). In the presence of PLP supplementation, the survival rate of the S-8Δ*pdxS* mutant was 38.58%, which was significantly lower (*P* < 0.01) than that of WT S-8 (86.09%); and the survival rate of S-8Δ*pdxT* mutant was 59.16%, which was also significantly lower (*P* < 0.05) than that of WT S-8 (Fig 5D). These results suggest that PLP synthases PdxS/PdxT are linked to resistance to osmotic stress and oxidative stress.

### Loss of *pdxS* and *pdxT* leads to morphological defects of *A*. *pleuropneumoniae*

**Fig 6:**
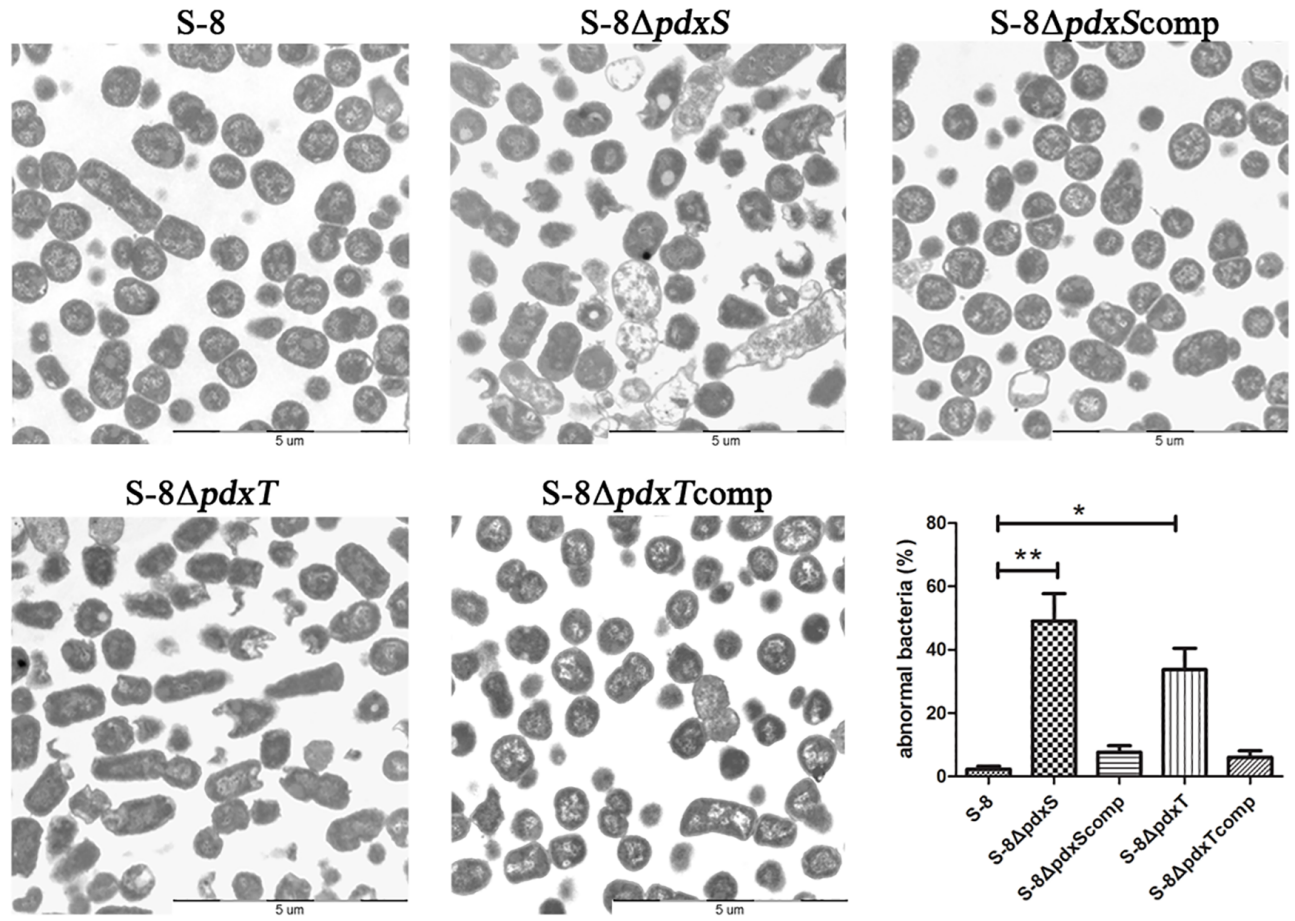
Transmission electron microscopy of *A*. *pleuropneumoniae* mutants in the absence of PLP. TEM of WT S-8, S-8*ΔpdxS*, S-8*ΔpdxT*, S-8Δ*pdxS*comp strain and S-8Δ*pdxT*comp strains in the mid-log phase in the absence of PLP were carried out. The cell morphology of the S-8Δ*pdxS* mutant and S-8Δ*pdxT* mutant exhibited irregular and aberrant shapes, showing holes and deep crater on their surface. S-8Δ*pdxS* also appeared partially lysed or swollen. Scale bar = 5μm.

The effects of PLP on the morphologies of WT S-8, S-8Δ*pdxS* mutant, S-8Δ*pdxT* mutant and complemented strains S-8Δ*pdxS*comp and S-8Δ*pdxT*comp were observed using transmission electron microscopy. These data showed that the morphologies of the five strains were similar in the presence of PLP supplementation, and were consistent with the normal morphology of coccobacilli (S4 Fig). However, in the absence of PLP, the S-8Δ*pdxS* mutant and S-8Δ*pdxT* mutant exhibited irregular and aberrant shapes, showing holes and deep craters on their surface. S-8Δ*pdxS* also appeared partially lysed or swollen (Fig 6). Approximately 49% of S-8Δ*pdxS* and 33.67% of S-8Δ*pdxT* cells were abnormal cells, whereas S-8, S-8Δ*pdxS*comp and S-8Δ*pdxT*comp strains exhibited only 2.3%, 7.7% and 6% of abnormal cells, respectively (Fig 6D). These data indicate that PLP production is required for maintaining normal cellular morphology of *A*. *pleuropneumoniae*.

### Loss of *pdxS* attenuates the virulence of *A*. *pleuropneumoniae* in the BALB/c mouse model

To address whether *pdxS* or *pdxT* deletion affected the virulence of *A*. *pleuropneumoniae*, BALB/c mice were inoculated intraperitoneally with WT S-8, S-8Δ*pdxS* mutant, and S-8Δ*pdxT* mutant strains at various doses, and LD50 values were determined. The LD_50_ value of the S-8Δ*pdxS* mutant was 2.51×10^8^ CFU, higher than 5.01×10^6^ CFU for WT S-8. The virulence of the S-8Δ*pdxS* mutant was 50.1-fold attenuated compared to that of WT S-8. Conversely, the LD_50_ value of the S-8Δ*pdxT* mutant (7.76×10^7^ CFU) was found to be only 15.5-fold lower than WT S-8. These results suggested that the loss of both *pdx* enzymes attenuates the pathogenicity of *A*. *pleuropneumoniae*, with a greater impact observed with the deletion of *pdxS*.

**Fig 7:**
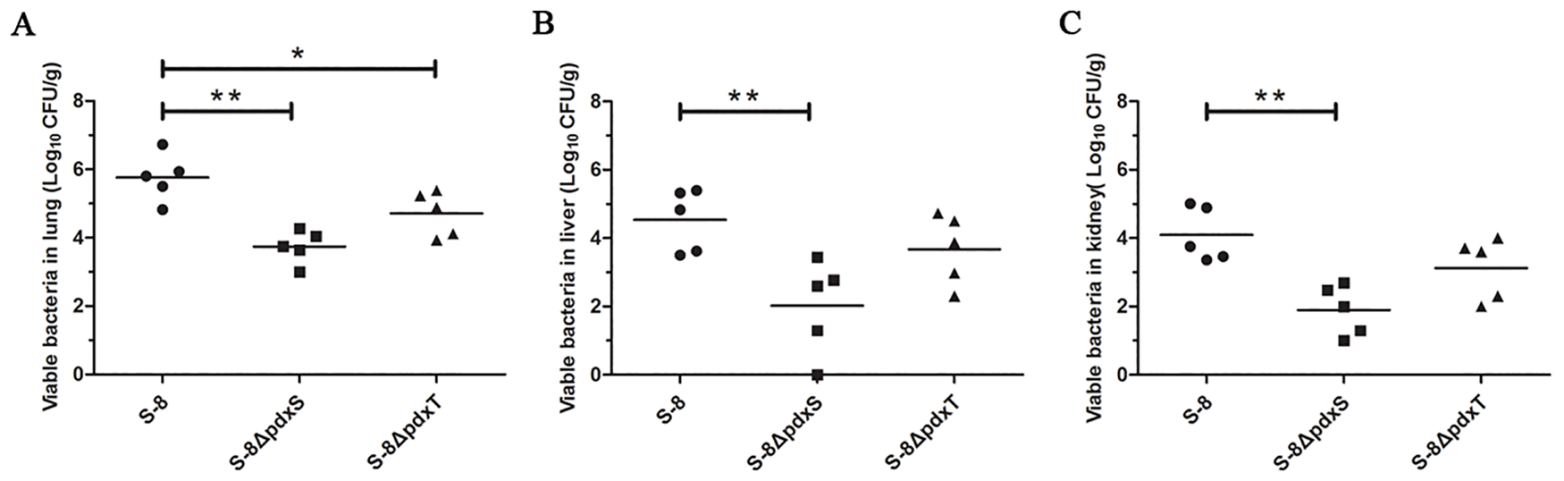
Bacterial loads in organs from BALB/c mice infected with *A*. *pleuropneumoniae* mutants. Mice were infected with WT S-8, S-8Δ*pdxS*, and S-8Δ*pdxT* mutant strains, and bacterial loads in (A) lung, (B) liver, (C) kidney examined 3 days post infection. The data shown are the means of bacterial colonies from five mice, and error bars indicate standard deviations. **, *p* < 0.01; *, *p* < 0.05.

The capacity of WT S-8, S-8Δ*pdxS* mutant, and S-8Δ*pdxT* mutant strains to colonize mice was then tested. The *A*. *pleuropneumoniae* load in tissues of systemically infected mice was determined by culturing the lungs, livers, and kidneys homogenates 3 days post-infection. As shown in Fig 7, the viable counts of the S-8Δ*pdxS* mutant in the lungs, livers and kidneys were significantly lower compared with those of WT S-8 (*P*<0.01). Similarly, significant differences (*P* < 0.05) in bacterial loads were also found between the WT S-8-inoculated mice and the S-8Δ*pdxT*-inoculated mice in lungs. In addition, the bacterial loads in the livers and kidneys of the S-8Δ*pdxT*-inoculated mice were decreased compared to the WT S-8-inoculated mice, although these differences were not statistically significant (Fig 7). Taken together, the results showed that the S-8Δ*pdxS* mutant of *A*. *pleuropneumoniae* displayed an impaired colonization ability in BALB/c mice.

## Discussion

In the *A*. *pleuropneumoniae* genome, only the adjacent *pdxS* and *pdxT* genes are annotated as belonging to the *pdx* family. Our results in the present study demonstrated that *A*. *pleuropneumoniae* synthesizes PLP via the DXP-independent pathway through the utilization of the two enzymes: the synthase subunit PdxS and the glutaminase subunit PdxT. In this study, we observed that respective disruption of the *pdxS* and *pdxT* genes rendered *A*. *pleuropneumoniae* auxotrophic for PLP (Fig 3), suggesting the importance of PLP biosynthesis in the growth of *A*. *pleuropneumoniae*. In addition, the results of the PLP synthase activity showed that both PdxS and PdxT proteins were required for PLP production *in vitro* in the presence of glutamine (Fig 4B and 4C). These findings are consistent with the previous observations in other bacteria.

In this study, the supplementation of exogenous ammonium in the growth broth complemented the defective growth rate of the S-8Δ*pdxT* mutant, but did not influence the defective growth rate of the S-8Δ*pdxS* mutant (Fig 4A). Similar results have also been reported in the *pdxT* deletion mutant in *S*. *pneumoniae* and *B*. *subtilis* whose defective growth rate could also be compensated in the presence of ammonium [9, 28], suggesting that the preliminary function of PdxT protein in PLP synthesis is producing ammonium from glutamine. Therefore, PdxS can produce PLP in the absence of PdxT when the glutamine is replaced by ammonium in the reaction (Fig 4C). These observations in this assay are in agreement with the proposed two-step model for PLP formation in previous studies [29, 30]. In this model, PdxT depends on PdxS to hydrolyze glutamine into ammonium, and PdxS utilizes ammonium for generating PLP by incorporation with other chemicals.

Intriguingly, we found that the PLP synthases PdxS/PdxT were implicated in the stress responses of *A*. *pleuropneumoniae*. To the best of our knowledge, this is the first study to show the relation between the PLP synthases PdxS/PdxT and stress response in bacteria. From the data presented here, it appeared that S-8Δ*pdxS* and S-8Δ*pdxT* mutants showed different responses *in vitro* to stress conditions compared with WT S-8. We found that *pdxS* and *pdxT* gene deletion caused increased susceptibility of *A*. *pleuropneumoniae* to H_2_O_2_-induced oxidative stress and NaCl-induced osmotic stress (Fig 5). Though these findings were not described in bacteria, PLP formation has been linked to stress tolerance in plants [31–33]. In *Arabidopsis thaliana*, disruption of PLP synthases lead to reduced plant size, slowed root growth and hypersensitivity to NaCl [31, 32]. In addition, PLP production in *A*. *thaliana* has also been associated with defense against cellular oxidative stress since *A*. *thaliana* mutants deficient in PLP synthase were shown to be highly sensitive to oxidative stresses [33]. Combining our findings presented here and previous work by others, it is highly likely that PLP synthases PdxS/PdxT play a vital role in osmotic stress and oxidative stress responses in *A*. *pleuropneumoniae*. However, the supplementation of exogenous PLP did not reverse the stress responses of the S-8Δ*pdxS* and S-8Δ*pdxT* mutants. We speculate that PdxS/PdxT may participate in other critical cellular processes involved in the biosynthesis of important enzymes associated with stress responses; however further investigations are required for confirmation.

Another objective of this study was to clarify whether PLP synthases PdxS/PdxT are essential for *A*. *pleuropneumoniae* pathogenicity in a mouse model. The results of this study indicated that the virulence and colonization of the S-8Δ*pdxS* and S-8Δ*pdxT* mutants were attenuated (Fig 7), although there were differences regarding the magnitude of attenuation, suggesting that the loss of the PLP biosynthesis pathway influences the pathogenicity of *A*. *pleuropneumoniae* in BALB/c mice. These findings are correlated with observations presented by other previous studies made in *M*. *tuberculosis*, *H*. *pylori* and *S*. *pneumoniae* [8, 9, 23]. We subsequently analyzed the probable reasons for attenuated virulence. It is likely that the PLP concentration in mice is insufficient for optimal growth of these *A*. *pleuropneumoniae* mutants *in vivo*. The PLP concentration in plasma of mice has been shown to fluctuate between 100 and 200 nM [34]. However, the addition of 100 nM of PLP to the broth did not significantly compensate the defective growth of the S-8Δ*pdxS* and S-8Δ*pdxT* mutants *in vitro* in our pilot experiments (data not shown), suggesting that the levels of PLP in mice may be too low to support the optimal growth of these mutants, which further affected the colonization and virulence of the S-8Δ*pdxS* and S-8Δ*pdxT* mutants. Another probability is the involvement of PLP in the biosynthesis of surface-exposed structures of *A*. *pleuropneumoniae*. Indeed, it has been shown that PLP biosynthesis is essential for the synthesis of surface-exposed structures involved in adhesion and colonization in some pathogenic bacteria, such as lipopolysaccharide (LPS) and flagella [23, 35, 36]. In this study, the S-8Δ*pdxS* and S-8Δ*pdxT* mutants exhibited aberrant shapes in the absence of PLP (Fig 6). However, whether this change directly affected the surface-exposed structures of *A*. *pleuropneumoniae* or then consequently affected the adhesion and colonization of *A*. *pleuropneumoniae*, needs further investigation.

Additionally, it is worth noting that the PLP biosynthesis pathway is present in various pathogenic bacteria, but is absent in humans and mammals [37, 38]. Therefore, the *de novo* pathway of PLP biosynthesis is of particular interest as novel potential drug targets for the therapy of bacterial infections. The data presented in this study demonstrated that disruption of PdxS/PdxT pathway of PLP biosynthesis inhibited the normal growth of *A*. *pleuropneumoniae*. This finding offers the new possibility of prevention and treatment of *A*. *pleuropneumoniae* infection which ravages the swine industry. In conclusion, the present study revealed the critical role of PLP synthases PdxS/PdxT in viability, stress tolerance, and virulence related to *A*. *pleuropneumoniae*. In addition, bacteria are also able to synthesize PLP by a salvage pathway [39], in which PLP is synthesized from other B6 vitamers. Future studies should include attempts to determine whether this pathway is present in *A*. *pleuropneumoniae* and its function in *A*. *pleuropneumoniae* pathogenicity.

## Supporting information

S1 Fig SDS—PAGE analysis of the recombinant PdxS and PdxT proteins purified with affinity chromatography. Recombinant PdxS (rPdxS) and PdxT (rPdxT) were separated by 12% SDS-PAGE. Lane M, molecular mass markers.
(TIF)

S2 Fig The relative transcription levels of selected genes. (A) Transcriptional levels of *pdxS* gene in WT S-8, S-8Δ*pdxS*, and S-8Δ*pdxS*comp strains. (B) Transcriptional levels of *pdxT* gene in WT S-8, S-8Δ*pdxT*, and S-8Δ*pdxT*comp strains. (C) Transcriptional levels of downstream genes of *pdxS* in WT S-8 and S-8Δ*pdxS* strains. (D) Transcriptional levels of downstream genes of *pdxT* in WT S-8 and S-8Δ*pdxT* strains.
(TIF)

S3 Fig Viable counts of *A*. *pleuropneumoniae* mutants exposed to oxidative and osmotic stresses. Overnight culture of WT S-8, S-8Δ*pdxS*, S-8Δ*pdxT*, S-8Δ*pdxScomp* strain and S-8Δ*pdxTcomp* strains were diluted into fresh BHI broth and grown to OD_600_ 0.8. Bacteria were then treated with 5 mM H_2_O_2_ in the absence (A) and presence (B) of PLP for 45 min, 0.4 M NaCl in the absence (C) and presence (D) of PLP for 45 min. Viable CFUs of *A*. *pleuropneumoniae* were counted. The data shown are the means of three independent assays.
(TIF)

S4 Fig Transmission electron microscopy of *A*. *pleuropneumoniae* mutants in the presence of PLP supplementation. TEM of WT S-8, S-8*ΔpdxS*, S-8*ΔpdxT*, S-8Δ*pdxS*comp strain and S-8Δ*pdxT*comp strains in the mid-log phase in the presence of PLP supplementation were carried out. The morphologies of the five strains were similar and were consistent with the normal morphology of coccobacilli.
(TIF)

S1 Table Characteristics of bacterial strains, plasmids, and primers used in this study. (DOCX)

S2 Table Primers used in qRT-PCR study. (DOCX)

## References

[1] Mooney S, Leuendorf JE, Hendrickson C, Hellmann H. Vitamin B6: a long known compound of surprising complexity. Molecules. 2009;14(1):329–51. 10.3390/molecules14010329 19145213 PMC6253932

[2] Eliot AC, Kirsch JF. Pyridoxal phosphate enzymes: mechanistic, structural, and evolutionary considerations. Annual review of biochemistry. 2004;73:383–415. 10.1146/annurev.biochem.73.011303.074021 15189147

[3] Percudani R, Peracchi A. A genomic overview of pyridoxal-phosphate-dependent enzymes. EMBO reports. 2003;4(9):850–4. 10.1038/sj.embor.embor914 12949584 PMC1326353

[4] Percudani R, Peracchi A. The B6 database: a tool for the description and classification of vitamin B6-dependent enzymatic activities and of the corresponding protein families. BMC bioinformatics. 2009;10:273 10.1186/1471-2105-10-273 19723314 PMC2748086

[5] Fitzpatrick TB, Amrhein N, Kappes B, Macheroux P, Tews I, Raschle T. Two independent routes of de novo vitamin B6 biosynthesis: not that different after all. The Biochemical journal. 2007;407(1):1–13. 10.1042/BJ20070765 17822383

[6] Mukherjee T, Hanes J, Tews I, Ealick SE, Begley TP. Pyridoxal phosphate: biosynthesis and catabolism. Biochimica et biophysica acta. 2011;1814(11):1585–96. 10.1016/j.bbapap.2011.06.018 21767669

[7] Tambasco-Studart M, Titiz O, Raschle T, Forster G, Amrhein N, Fitzpatrick TB. Vitamin B6 biosynthesis in higher plants. Proceedings of the National Academy of Sciences of the United States of America. 2005;102(38):13687–92. 10.1073/pnas.0506228102 16157873 PMC1224648

[8] Dick T, Manjunatha U, Kappes B, Gengenbacher M. Vitamin B6 biosynthesis is essential for survival and virulence of *Mycobacterium tuberculosis*. Molecular microbiology. 2010;78(4):980–8. 10.1111/j.1365-2958.2010.07381.x 20815826

[9] El Qaidi S, Yang J, Zhang JR, Metzger DW, Bai G. The vitamin B(6) biosynthesis pathway in *Streptococcus pneumoniae* is controlled by pyridoxal 5'-phosphate and the transcription factor PdxR and has an impact on ear infection. Journal of bacteriology. 2013;195(10):2187–96. 10.1128/JB.00041-13 23475965 PMC3650526

[10] Raschle T, Amrhein N, Fitzpatrick TB. On the two components of pyridoxal 5'-phosphate synthase from *Bacillus subtilis*. The Journal of biological chemistry. 2005;280(37):32291–300. 10.1074/jbc.M501356200 16030023

[11] Chiers K, De Waele T, Pasmans F, Ducatelle R, Haesebrouck F. Virulence factors of *Actinobacillus pleuropneumoniae* involved in colonization, persistence and induction of lesions in its porcine host. Veterinary research. 2010;41(5):65 10.1051/vetres/2010037 20546697 PMC2899255

[12] Bosse JT, Janson H, Sheehan BJ, Beddek AJ, Rycroft AN, Kroll JS, et al *Actinobacillus pleuropneumoniae*: pathobiology and pathogenesis of infection. Microbes and infection / Institut Pasteur. 2002;4(2):225–35. 10.1016/s1286-4579(01)01534-9 11880056

[13] Li G, Xie F, Zhang Y, Wang C. Draft genome sequence of *Actinobacillus pleuropneumoniae* serotype 7 strain S-8. Journal of bacteriology. 2012;194(23):6606–7. 10.1128/JB.01650-12 23144372 PMC3497496

[14] Xie F, Zhang Y, Li G, Zhou L, Liu S, Wang C. The ClpP protease is required for the stress tolerance and biofilm formation in *Actinobacillus pleuropneumoniae*. PloS one. 2013;8(1):e53600 10.1371/journal.pone.0053600 23326465 PMC3543445

[15] Wagner TK, Mulks MH. A subset of *Actinobacillus pleuropneumoniae* in vivo induced promoters respond to branched-chain aminoacid limitation. FEMS Immunology and Medical Microbiology. 2006; 48(2):192–204. 10.1111/j.1574-695X.2006.00147.x 16995880

[16] Chen L, Dang G, Deng X, Cao J, Yu S, Wu D, et al Characterization of a novel exported esterase Rv3036c from *Mycobacterium tuberculosis*. Protein expression and purification. 2014;104:50–6. 10.1016/j.pep.2014.09.003 25224799

[17] Strohmeier M, Raschle T, Mazurkiewicz J, Rippe K, Sinning I, Fitzpatrick TB, et al Structure of a bacterial pyridoxal 5'-phosphate synthase complex. Proceedings of the National Academy of Sciences of the United States of America. 2006;103(51):19284–9. 10.1073/pnas.0604950103 17159152 PMC1748218

[18] Baltes N, Tonpitak W, Hennig-Pauka I, Gruber AD, Gerlach GF. *Actinobacillus pleuropneumoniae* serotype 7 siderophore receptor FhuA is not required for virulence. FEMS microbiology letters. 2003;220(1):41–8. 12644226 10.1016/S0378-1097(03)00064-8

[19] Dehio C, Meyer M. Maintenance of broad-host-range incompatibility group P and group Q plasmids and transposition of Tn5 in *Bartonella henselae* following conjugal plasmid transfer from *Escherichia coli*. Journal of bacteriology. 1997;179(2):538–40. 8990308 10.1128/jb.179.2.538-540.1997 PMC178726

[20] Oswald W, Tonpitak W, Ohrt G, Gerlach G. A single-step transconjugation system for the introduction of unmarked deletions into *Actinobacillus pleuropneumoniae* serotype 7 using a sucrose sensitivity marker. FEMS microbiology letters. 1999;179(1):153–60. 10481100 10.1111/j.1574-6968.1999.tb08721.x

[21] Willson PJ, Albritton WL, Slaney L, Setlow JK. Characterization of a multiple antibiotic resistance plasmid from *Haemophilus ducreyi*. Antimicrobial Agents and Chemotherapy. 1989; 33(9):1627–30. 2684012 10.1128/aac.33.9.1627 PMC172718

[22] Nielsen KK, Boye M. Real-time quantitative reverse transcription-PCR analysis of expression stability of *Actinobacillus pleuropneumoniae* housekeeping genes during in vitro growth under iron-depleted conditions. Applied and environmental microbiology. 2005;71(6):2949–54. 10.1128/AEM.71.6.2949-2954.2005 15932989 PMC1151834

[23] Grubman A, Phillips A, Thibonnier M, Kaparakis-Liaskos M, Johnson C, Thiberge JM, et al Vitamin B6 is required for full motility and virulence in *Helicobacter pylori*. mBio. 2010;1(3). 10.1128/mBio.00112-10 PMC3000542 21151756

[24] Chiang CH, Huang WF, Huang LP, Lin SF, Yang WJ. Immunogenicity and protective efficacy of ApxIA and ApxIIA DNA vaccine against *Actinobacillus pleuropneumoniae* lethal challenge in murine model. Vaccine. 2009;27(34):4565–70. 10.1016/j.vaccine.2009.05.058 19520199

[25] Seo KW, Kim SH, Park J, Son Y, Yoo HS, Lee KY, et al Nasal immunization with major epitope-containing ApxIIA toxin fragment induces protective immunity against challenge infection with *Actinobacillus pleuropneumoniae* in a murine model. Veterinary immunology and immunopathology. 2013;151(1–2):102–12. 10.1016/j.vetimm.2012.10.011 23200821

[26] Nemzek JA, Xiao HY, Minard AE, Bolgos GL, Remick DG. Humane endpoints in shock research. Shock. 2004;21(1):17–25. Epub 2003/12/17. 10.1097/01.shk.0000101667.49265.fd 14676679

[27] Reed L, Muench H. A simple method of estimating fifty per cent endpoints. Am. J. Epidemiol.; 1938 p. 493–7.

[28] Belitsky BR. Physical and enzymological interaction of *Bacillus subtilis* proteins required for de novo pyridoxal 5'-phosphate biosynthesis. Journal of bacteriology. 2004;186(4):1191–6. 10.1128/JB.186.4.1191-1196.2004 14762015 PMC344226

[29] Neuwirth M, Flicker K, Strohmeier M, Tews I, Macheroux P. Thermodynamic characterization of the protein-protein interaction in the heteromeric *Bacillus subtilis* pyridoxalphosphate synthase. Biochemistry. 2007;46(17):5131–9. 10.1021/bi602602x 17408246

[30] Wallner S, Neuwirth M, Flicker K, Tews I, Macheroux P. Dissection of contributions from invariant amino acids to complex formation and catalysis in the heteromeric pyridoxal 5-phosphate synthase complex from *Bacillus subtilis*. Biochemistry. 2009;48(9):1928–35. 10.1021/bi801887r 19152323

[31] Gonzalez E, Danehower D, Daub ME. Vitamer levels, stress response, enzyme activity, and gene regulation of Arabidopsis lines mutant in the pyridoxine/pyridoxamine 5'-phosphate oxidase (PDX3) and the pyridoxal kinase (SOS4) genes involved in the vitamin B6 salvage pathway. Plant physiology. 2007;145(3):985–96. 10.1104/pp.107.105189 17873088 PMC2048783

[32] Denslow SA, Rueschhoff EE, Daub ME. Regulation of the *Arabidopsis thaliana* vitamin B6 biosynthesis genes by abiotic stress. Plant physiology and biochemistry: PPB / Societe francaise de physiologie vegetale. 2007;45(2):152–61. 10.1016/j.plaphy.2007.01.007 17344055

[33] Chen H, Xiong L. Pyridoxine is required for post-embryonic root development and tolerance to osmotic and oxidative stresses. The Plant journal: for cell and molecular biology. 2005;44(3):396–408. 16236150 10.1111/j.1365-313X.2005.02538.x

[34] Vandekamp JL, Westrick JA, Smolen A. B-6 Vitamer Concentrations in Mouse Plasma, Erythrocytes and Tissues. Nutr Res. 1995;15(3):415–22.

[35] Sheng H, Lim JY, Watkins MK, Minnich SA, Hovde CJ. Characterization of an *Escherichia coli* O157:H7 O-antigen deletion mutant and effect of the deletion on bacterial persistence in the mouse intestine and colonization at the bovine terminal rectal mucosa. Applied and environmental microbiology. 2008;74(16):5015–22. 10.1128/AEM.00743-08 18552194 PMC2519267

[36] Asakura H, Hashii N, Uema M, Kawasaki N, Sugita-Konishi Y, Igimi S, et al *Campylobacter jejuni pdxA* affects flagellum-mediated motility to alter host colonization. PloS one. 2013;8(8):e70418 10.1371/journal.pone.0070418 23936426 PMC3735588

[37] Knockel J, Muller IB, Butzloff S, Bergmann B, Walter RD, Wrenger C. The antioxidative effect of de novo generated vitamin B6 in *Plasmodium falciparum* validated by protein interference. The Biochemical journal. 2012;443(2):397–405. 10.1042/BJ20111542 22242896

[38] Ma W, Cao W, Zhang B, Chen K, Liu Q, Li Y, et al Engineering a pyridoxal 5'-phosphate supply for cadaverine production by using Escherichia coli whole-cell biocatalysis. Scientific reports. 2015;5:15630 10.1038/srep15630 26490441 PMC4614675

[39] Yang Y, Tsui HC, Man TK, Winkler ME. Identification and function of the pdxY gene, which encodes a novel pyridoxal kinase involved in the salvage pathway of pyridoxal 5'-phosphate biosynthesis in *Escherichia coli* K-12. Journal of bacteriology. 1998;180(7):1814–21. 9537380 10.1128/jb.180.7.1814-1821.1998 PMC107095

