# Supplementary figures and images for: Pyridoxal phosphate synthases PdxS/PdxT are required for Actinobacillus pleuropneumoniae viability, stress tolerance and virulence
Source: PLoS One. 2017 Apr 27;12(4):e0176374. doi: 10.1371/journal.pone.0176374 (PMC5407770; doi:10.1371/journal.pone.0176374)

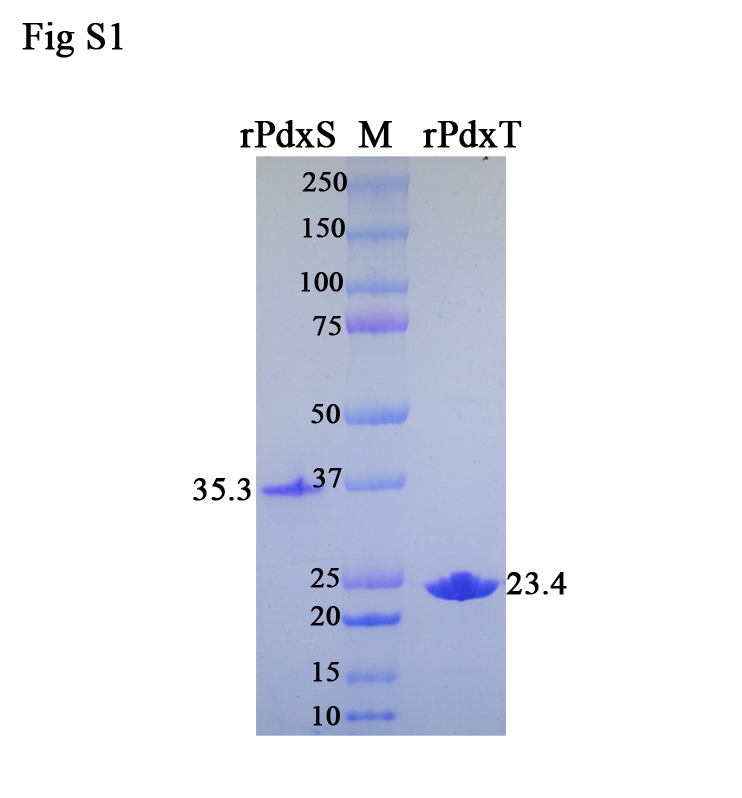

Supplement: S1 Fig — Recombinant PdxS (rPdxS) and PdxT (rPdxT) were separated by 12% SDS-PAGE. Lane M, molecular mass markers. (TIF) [file pone.0176374.s001.tif]

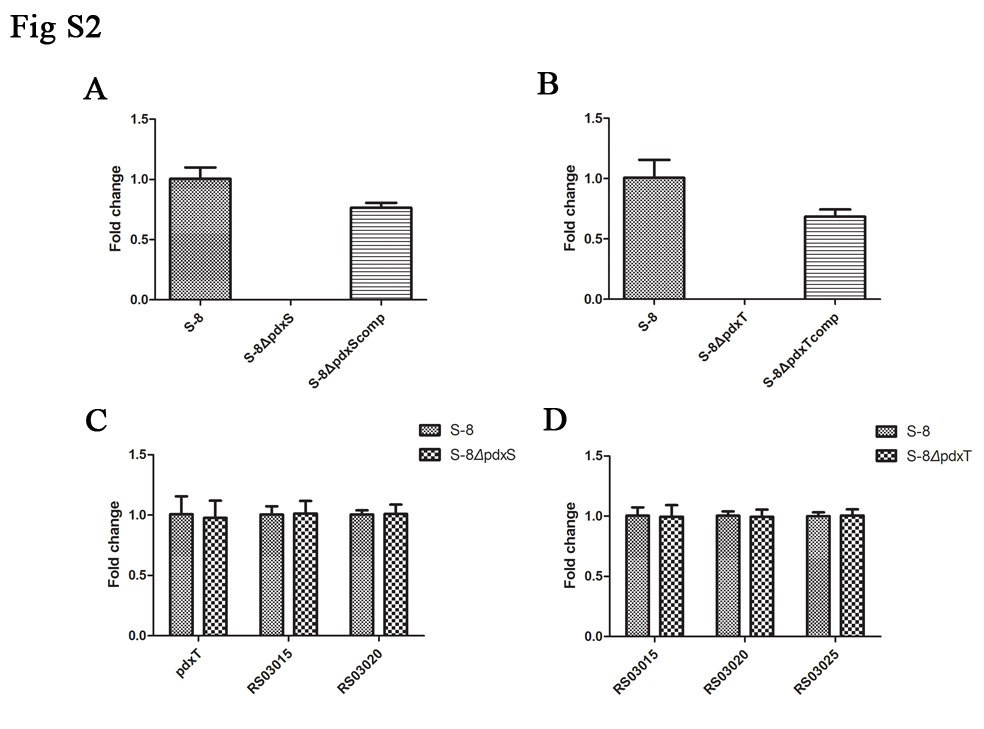

Supplement: S2 Fig — (A) Transcriptional levels of pdxS gene in WT S-8, S-8ΔpdxS, and S-8ΔpdxScomp strains. (B) Transcriptional levels of pdxT gene in WT S-8, S-8ΔpdxT, and S-8ΔpdxTcomp strains. (C) Transcriptional levels of downstream genes of pdxS in WT S-8 and S-8ΔpdxS strains. (D) Transcriptional levels of downstream genes of pdxT in WT S-8 and S-8ΔpdxT strains. (TIF) [file pone.0176374.s002.tif]

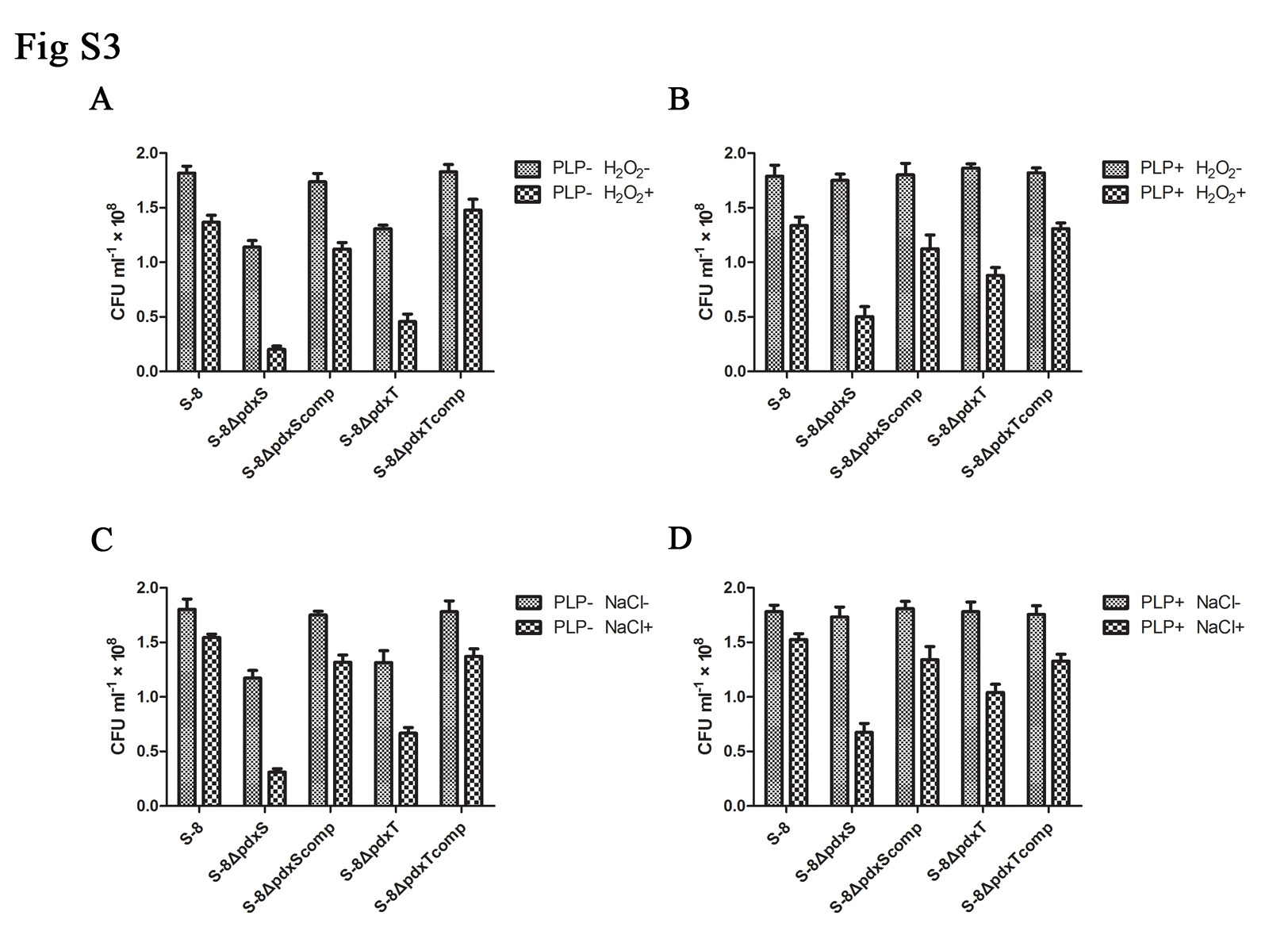

Supplement: S3 Fig — Overnight culture of WT S-8, S-8ΔpdxS, S-8ΔpdxT, S-8ΔpdxScomp strain and S-8ΔpdxTcomp strains were diluted into fresh BHI broth and grown to OD600 0.8. Bacteria were then treated with 5 mM H2O2 in the absence (A) and presence (B) of PLP for 45 min, 0.4 M NaCl in the absence (C) and presence (D) of PLP for 45 min. Viable CFUs of A. pleuropneumoniae were counted. The data shown are the means of three independent assays. (TIF) [file pone.0176374.s003.tif]

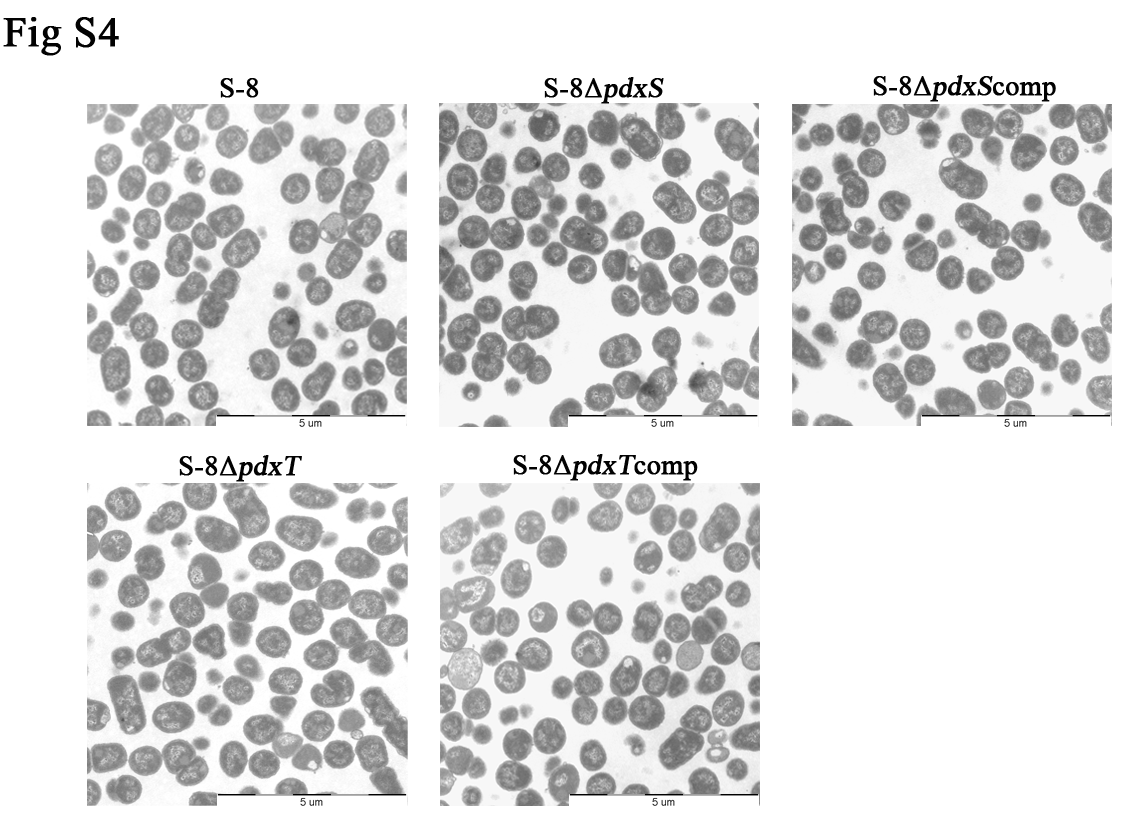

Supplement: S4 Fig — TEM of WT S-8, S-8ΔpdxS, S-8ΔpdxT, S-8ΔpdxScomp strain and S-8ΔpdxTcomp strains in the mid-log phase in the presence of PLP supplementation were carried out. The morphologies of the five strains were similar and were consistent with the normal morphology of coccobacilli. (TIF) [file pone.0176374.s004.tif]
