# Supplementary material for: Pyridoxal phosphate synthases PdxS/PdxT are required for Actinobacillus pleuropneumoniae viability, stress tolerance and virulence
Source: PLoS One. 2017 Apr 27;12(4):e0176374. doi: 10.1371/journal.pone.0176374 (PMC5407770; doi:10.1371/journal.pone.0176374)
Supplement: S1 Table — (DOCX) [file pone.0176374.s005.docx]

**Table S1**

**Characteristics of bacterial strains, plasmids, and primers used in this study**

| Strains, plasmids, and primers | Characteristics or sequence | Source or  reference |
| --- | --- | --- |
| Strains | | |
| *A.pleuropneumoniae* S-8 | *A. pleuropneumoniae* serovar 7 clinical isolate from the lung of a dead pig with pleuropneumonia in northern China | [14] |
| *A.pleuropneumoniae* S-8Δ*pdxS* | Unmarked *pdxS* gene knockout mutant of *A. pleuropneumoniae* S-8 | This work |
| *A.pleuropneumoniae*  S-8Δ*pdxT* | Unmarked *pdxT* gene knockout mutant of *A. pleuropneumoniae* S-8 | This work |
| *E. coli* β2155 | *thrB1004 pro thi strA hsdS lac*Z△M15 (F' *lacZ*△M15 *lacl*^q^ *traD36 proA*^+^ *proB*^+^ )*△dap* :: *erm* (Erm^r^))*recA* :: *RPA-2-tet*(Tc^r^)::Mu-km (Km^r^) λ*pir* | [19] |
| *E. coli* DH5α | *E. coli* strain used for cloning | Lab stock |
| *E. coli* BL21(DE3) | *E. coli* strain used for expression | Novagen |
| Plasmids | | |
| pEMOC2 | Conjugative vector based on pBluescript SK with mobRP4, polycloning site, *Cm^r^*, and transcriptional fusion of the *omlA* promoter with the *sacB* gene | Accession no. AJ868288  [18] |
| pEM*△pdxS* | Conjugative vector pEMOC2 with a 487bp deletion in the *pdxS* gene which have a 1.2-kb upstream fragment and 1.3-kb downstream fragment | This work |
| pEM*△pdxT* | Conjugative vector pEMOC2 with a 491bp deletion in the *pdxT* gene which have a 1.3-kb upstream fragment and 1.3-kb downstream fragment | This work |
| pLS88 | Broad-host-range shuttle vector from *Haemophilus ducreyi*; Str^r^ Sm^r^ Km^r^ | [21] |
| pLpdxS | pLS88 with a PCR-derived insert containing the *pdxS* gene | This work |
| pLpdxT | pLS88 with a PCR-derived insert containing the *pdxT* gene | This work |
| pET22b(+) | His tag expression vector; Ap^r^ | Novagen |
| pET22b-pdxS | pET22b(+) carrying *A. pleuropneumoniae* *pdxS* ORF | This work |
| pET22b-pdxT | pET22b(+) carrying *A. pleuropneumoniae* *pdxT* ORF | This work |
| Primers | | |
| SUF | 5' GCGTCGACGCGGTAGATTTTTCTCATTCG 3', upstream primer with internal SalI site (underlined) | This work |
| SUR | 5' CAGTTCGTC**TGCATTTGA**GCCATTCCAC 3', downstream primer with reverse complement sequence(underlined) of sequence in bold from primer SDF | This work |
| SDF | 5' TCAAATGCA**GACGAACTG**ATGACCGAAGC 3', upstream primer with reverse complement sequence(underlined) of sequence in bold from primer SUR | This work |
| SDR | 5' TTGCGGCCGCAGCGGGCAAAGTACAACG 3', downstream primer with internal NotI site (underlined) | This work |
| TUF | 5' GCGTCGACTGGCACACTTTTTCCAACG 3', upstream primer with internal SalI site (underlined) | This work |
| TUR | 5' CAAGCCAGT**GTTCATTAC**CAACCACGCTC 3', downstream primer with reverse complement sequence(underlined) of sequence in bold from primer TDF | This work |
| TDF | 5' GTAATGAAC**ACTGGCTTG**TGCGTTTCATC 3', upstream primer with reverse complement sequence(underlined) of sequence in bold from primer TUR | This work |
| TDR | 5' TTGCGGCCGCGCCGACCAAGATCACGAAG 3', downstream primer with internal NotI site (underlined) | This work |
| SCF | 5' GGAATTCAAGCAGCTTTCTACGCCCG 3', upstream primer for identification of S-8△*pdxS* mutant and construction of the complemented strains S-8△*pdxS*comp and S-8△*pdxT*comp | This work |
| SCR | 5' CGAGCTCAAACCGCCCCCTGTAATG 3', downstream primer for identification of S-8△*pdxS* mutant and construction of the complemented strain S-8△*pdxS*comp | This work |
| pR | 5' TCGTTCAT**ATGAATTT**CCTTTTTATTGATGTG 3', downstream primer with reverse complement sequence(underlined) of sequence in bold from primer TCF and construction of the complemented strain S-8△*pdxT*comp | This work |
| TCF | 5' AAATTCAT**ATGAACGA**TTATACAAAATATACCATAG 3', upstream primer with reverse complement sequence(underlined) of sequence in bold from primer pR and construction of the complemented strain S-8△*pdxT*comp | This work |
| TCR | 5' CGAGCTCAAAATGCAATTCGATCTAAGG 3', downstream primer for construction of the complemented strain S-8△*pdxT*comp | This work |
| SF | 5'-GATCCATATGATGACCAAAATTTTAGGTTCGG-3', upstream primer with internal NdeI site (underlined) | This work |
| SR | 5'-GATCCTCGAGCCAACCACGCTCTTGCATAC-3', downstream primer with internal XhoI site (underlined) | This work |
| TF | 5'-GATCCATATGATGAACGATTATACAAAATATACCATAG-3', upstream primer with internal NdeI site (underlined) | This work |
| TR | 5'-GATCCTCGAGCTCTTTTAGCTGTTGTAGAAACAATTC-3', downstream primer with internal XhoI site (underlined) | This work |
