# Supplementary material for: Pyridoxal phosphate synthases PdxS/PdxT are required for Actinobacillus pleuropneumoniae viability, stress tolerance and virulence
Source: PLoS One. 2017 Apr 27;12(4):e0176374. doi: 10.1371/journal.pone.0176374 (PMC5407770; doi:10.1371/journal.pone.0176374)
Supplement: S2 Table — (DOCX) [file pone.0176374.s006.docx]

**Table S2**

**Primers used in qRT-PCR study**

| Primer | Sequences (5’–3’) | Target | Source or reference |
| --- | --- | --- | --- |
| RTrecF | TATGCCGAGATTCTTGCTCA | *recF* | [22] |
| RTrecR | AATTTAAGCTGCCCACGAGA |  |  |
| RTSF | CTATTGTGCGTGAAGTGATGGA | *pdxS* | This work |
| RTSR | AGTGCTTCGCCTAAATCTCGG |  |  |
| RTTF | AGCGGTTTATTTCAGGCACTC | *pdxT* | This work |
| RTTR | CGCCAGCACTTCTACCTCACT |  |  |
| RT03015F | TTATCGGCGAAGGCTGGAATC | *APL_RS03015* | This work |
| RT03015R | TAAACGGCGTAAGCGGCTATG |  |  |
| RT03020F | TTACGACATTCAATTACGTGCTT | *APL_RS03020* | This work |
| RT03020R | TTCGTCCGCTTCATCCAATAC |  |  |
| RT03025F | ATGACAGTGCGATTGATGCC | *APL_RS03025* | This work |
| RT03025R | CCAATTCGTTGAAATATAGCCA |  |  |
